# Evaluation of Real-Time Intracochlear Electrocochleography for Guiding Cochlear Implant Electrode Array Position

**DOI:** 10.3390/jcm12237409

**Published:** 2023-11-29

**Authors:** Rachel Scheperle, Christine Etler, Jacob Oleson, Camille Dunn, Rustin Kashani, Alexander Claussen, Bruce J. Gantz, Marlan R. Hansen

**Affiliations:** 1 Department of Otolaryngology-Head and Neck Surgery, University of Iowa Hospitals and Clinics, Iowa City, IA 52242, USA; christine-etler@uiowa.edu (C.E.); camille-dunn@uiowa.edu (C.D.); rustin-kashani@uiowa.edu (R.K.); alexander-claussen@uiowa.edu (A.C.); bruce-gantz@uiowa.edu (B.J.G.); marlan-hansen@uiowa.edu (M.R.H.); 2 Department of Biostatistics, University of Iowa, Iowa City, IA 52242, USA; 3 Department of Neurosurgery, University of Iowa Hospitals and Clinics, Iowa City, IA 52242, USA; 4 Department of Molecular Physiology and Biophysics, University of Iowa Hospitals and Clinics, Iowa City, IA 52242, USA

**Keywords:** electrocochleography, cochlear implant, hearing preservation

## Abstract

This study evaluates intracochlear electrocochleography (ECochG) for real-time monitoring during cochlear implantation. One aim tested whether adjusting the recording electrode site would help differentiate between atraumatic and traumatic ECochG amplitude decrements. A second aim assessed whether associations between ECochG amplitude decrements and post-operative hearing loss were weaker when considering hearing sensitivity at the ECochG stimulus frequency compared to a broader frequency range. Eleven adult cochlear implant recipients who were candidates for electro-acoustic stimulation participated. Single-frequency (500-Hz) ECochG was performed during cochlear implantation; the amplitude of the first harmonic of the difference waveform was considered. Post-operative hearing preservation at 500 Hz ranged from 0 to 94%. The expected relationship between ECochG amplitude decrements and hearing preservation was observed, though the trend was not statistically significant, and predictions were grossly inaccurate for two participants. Associations did not improve when considering alternative recording sites or hearing sensitivity two octaves above the ECochG stimulus frequency. Intracochlear location of a moving recording electrode is a known confound to real-time interpretation of ECochG amplitude fluctuations, which was illustrated by the strength of the correlation with ECochG amplitude decrements. Multiple factors contribute to ECochG amplitude patterns and to hearing preservation; these results highlight the confounding influence of intracochlear recording electrode location on the ECochG.

## 1. Introduction

Reducing intracochlear trauma during cochlear implantation is desirable not only to optimize acoustic hearing preservation but also to maximize the general benefit from electrical stimulation [1,2,3]. Surgical approaches have evolved alongside electrode array designs, with the intent of minimizing destructive disturbance to the peripheral auditory system [4,5]. Using the patient’s anatomy to guide the selection of a specific electrode array and to influence surgical plans regarding insertion depth [6,7,8] has become standard in some centers, including ours, facilitated by the expansion of electrode array options and the availability of image analysis tools. Robotic devices are being developed to improve surgical precision, accuracy, and control (e.g., reviewed by De Seta and colleagues [9]). Another advancement is the use of intraoperative monitoring of acoustically evoked potentials to provide real-time physiological feedback during positioning of the electrode array (reviewed by Trecca and colleagues [10]). This center routinely uses a combination of atraumatic approaches to increase the likelihood of maintaining structural and functional integrity of the auditory periphery; this project evaluates the use of electrocochleography (ECochG) to guide electrode array positioning in individuals with sufficient residual hearing pre-operatively to be considered candidates for electro-acoustic stimulation.

Electrocochleography is well suited for monitoring intracochlear trauma from electrode array insertion because the contributing physiological generators are peripheral, including cochlear hair cells (inner and outer) and auditory afferent fibers. Components of the composite ECochG include onset/offset responses (compound action potential; CAP), an ongoing, sustained response (summating potential; SP, and auditory nerve sustained potential; ANSP), and an ongoing, frequency-following response (cochlear microphonic; CM, and auditory nerve neurophonic; ANN; [11,12,13]). For real-time monitoring applications, the ongoing, frequency-following response is typically used due to a larger response and better signal-to-noise ratio [14,15,16] (however, see [17,18] for evidence supporting the use of the CAP). Although some differences exist across investigations, a high-level, low-frequency (often 500 Hz) tone burst presented with alternating starting polarity is frequently used for real-time monitoring. The amplitude of the first harmonic calculated from a fast Fourier transform (FFT) performed on the subtracted waveform, which is dominated by the CM/hair cell contributions at high intensities and low frequencies [12], represents the most frequently used response metric.

Sensitivity of the ECochG response to cochlear trauma has been directly observed in animals when blunt force impact has been intentional, and injury to the osseous spiral lamina, basilar membrane, or spiral ligament is confirmed by histology [17,19,20]. Amplitude decrements following mechanical insults occur in both normal-hearing animal models and models with pre-surgical hearing loss. Notably, response decrements can be graded, relating to the degree of damage, and some are reversible [17,19], suggesting that prevention of permanent or worsening injury is possible.

In humans, careful measures are taken to avoid traumatic injury to residual elements of the inner ear during cochlear implantation. Even so, hearing loss shortly after the surgery ranging from partial to complete implies traumatic events of varying degrees [21]. Despite a strong hypothetical framework, the expected relationship (i.e., poorer hearing preservation in cases when ECochG amplitude declined during insertion) has been observed in some but not all data sets, and a substantial amount of unexplained variability exists [16,22,23,24,25,26,27]. Scalar translocation is one of the more extreme traumatic events that can occur during electrode array insertion. This irreversible injury can be visualized in humans with post-operative imaging, and is associated with greater loss of acoustic hearing than insertions that remain within the scala tympani [26]. Even so, the sensitivity of ECochG amplitude decrements during electrode insertion to detect translocations is lower than anticipated [26,28].

Although interest in intraoperative ECochG monitoring for optimizing electrode placement remains high, it is also clear that current approaches complicate real-time interpretation. A primary example is the confound introduced by using the electrode array for recording the ECochG. An intracochlear recording site is desirable because it generally measures a substantially larger signal compared to an extracochlear (often round window) recording site [14,29,30]. A larger signal and a better signal-to-noise ratio is advantageous for speed: critical for real-time monitoring applications during a relatively short procedure. Moreover, the systems developed by cochlear implant (CI) manufacturers that integrate acoustic stimulation with recording from the intracochlear electrode array simplify the equipment set-up and streamline the process (Advanced Bionics [31]; Cochlear [15]; MedEl [32]). The ability to use the electrode array for recording is considered a significant advancement, as the measurement tool is now more widely accessible. The primary disadvantage is a more complicated real-time interpretation of ECochG amplitude changes due to the moving recording site.

Despite the proximity to the response generators that an intracochlear electrode affords, it is important to remember that the ECochG is a field potential, and the amplitude is the integration of all contributing generators, weighted by nearness and orientation to the recording site. Generator contributions are controlled by the amplitude and phase of basilar membrane/tectorial membrane displacement, which has both excitatory and inhibitory motion occurring simultaneously across its length [33]. Specifically, the motion near the peak region can be out of phase compared to the broader in-phase tail region. Some generator contributions will be cancelled at locations within the cochlear duct as both positive and negative receptor potentials are simultaneously produced (see [30,34] for additional explanation).

Incorporating other metrics derived from the ECochG to consider along with amplitude decrements has been proposed to help differentiate which decrements reflect trauma from ones that do not. Adding the rate of amplitude change to the size of the amplitude decrement improves predictions [35]; however, a criterion slope value may not apply to all recording systems given different averaging and update rates. Considering the phase or latency information from the ECochG waveform has also been investigated [30,35,36]. Incorporating a component with greater neural contributions, such as ANN or CAP, has also been suggested and attempted [30,37].

While many of these approaches are promising, they are not yet available with manufacturer systems or are not available to be used in real time as feedback. Evoking the ECochG using multiple frequencies and comparing associated response patterns is currently feasible [24,25,38,39]. While theoretical advantages exist, whether or not those advantages improve real-time interpretation (and ultimately post-operative outcomes) remains unknown.

The purpose of this study was to further investigate the real-time interpretation of single-frequency ECochG amplitude decrements during electrode array insertion. The overarching goal was to evaluate whether ECochG amplitude patterns were predictive of post-operative hearing loss in a setting where feedback was available to the surgeon. The first aim was to explore whether a monitoring strategy that included the opportunity to adjust the recording electrode in real time would improve interpretation of an amplitude decrement. It was hypothesized that recording similarly diminished amplitudes at a more basal site would confirm results at the more apical site, indicating trauma, while recording larger amplitudes at a more basal site would signify better hearing preservation outcomes than predicted by the apical recording. Giardina and colleagues [30] suggested changing the recording electrode throughout the measurement to keep the recording site stable at a basal location. In our experience, a response from electrodes positioned in the base is often not measurable or unreliable due to low amplitude relative to the noise floor at the initiation of a recording. Moreover, adjusting the recording electrode requires a pause in the surgical procedure, which lengthens surgical time. Here, our initial attempts to use multiple recording electrodes within the context of practical constraints are described.

The second aim considered whether the single-frequency ECochG would be most predictive of hearing sensitivity in the same frequency region or across a broader frequency range. Traveling-wave patterns initiated by suprathreshold, low-frequency tone-burst stimuli used to evoke the ECochG are broader than traveling-wave patterns initiated by threshold-level, low-frequency tonal stimuli used to assess hearing sensitivity. It was hypothesized that correlations between ECochG amplitude patterns and hearing preservation would be stronger when considering hearing sensitivity across a range of frequencies (i.e., at and higher than the ECochG stimulus frequency) compared to hearing sensitivity matched to the ECochG stimulus frequency due to basilar membrane displacement in the basal tail of the traveling wave.

Finally, the results were also used to further illustrate the confounding impact of the intracochlear location of the recording electrode on ECochG amplitude decrements. It was predicted that unanticipated hearing preservation outcomes would be observed in cases for which the tonotopic frequency associated with the intracochlear location of the apical recording electrode is nearer to the ECochG stimulus frequency.

## 2. Materials and Methods

All procedures were performed in accordance with ethical standards for human research and approved by the University of Iowa Hospitals and Clinics (UIHC) Institutional Review Board (IRB 201805740 or 202210440). Informed consent was obtained from all subjects involved in the study.

**Figure 1:**
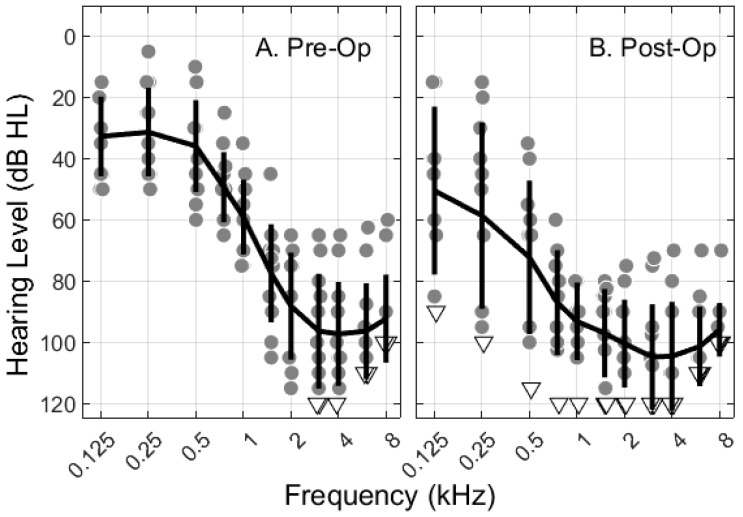
Behavioral pure-tone audiometric hearing thresholds before (**A**) and after (**B**) cochlear implant surgery. Individual thresholds are indicated with gray circles. Down-pointing, open triangles were positioned 5 dB greater than the maximum output of the audiometer when no response was observed at the audiometer limits, which were also included in the mean/standard deviation calculations. Solid black lines pass through the means with vertical bars extending ±1 standard deviation. Symbols are jittered around frequency for visualization of overlapping points.

Adult patients at UIHC who were deemed CI candidates, who met Med-El electric-acoustic stimulation (EAS) candidacy criteria (i.e., 18 years or older, low-frequency hearing ranging from normal to moderate sensorineural hearing loss, and severe to profound high-frequency sensorineural hearing loss; see Figure 1A), and who selected Med-El devices (Med-El Corporation, Innsbruck, Austria) in the period between January 2022 and April 2023 were considered for participation. Initial participants in the earliest pilot phase of testing were excluded due to inconsistency of methods (e.g., variable ECochG stimulus parameters) or lack of imaging data. Fourteen consenting individuals were implanted between September 2022 and April 2023. Two were excluded due to hardware problems. One was excluded due to premature opening of the round window during removal of scar tissue. Information about the 11 included participants is provided in Table 1.

Pre- and post-operative computed tomography (CT) imaging has become a standard component of the CI evaluation and follow-up process at this institution. A subset of scans was photon-counting. OTOPLAN V.02 or V.03 software (CAScination AG, Bern, Switzerland) was used for cochlear measurements and parameter estimates (see Table 1). Pre-operative estimates are used clinically to influence electrode array selection, and post-operative estimates can be used to influence programming decisions.

The “electrode visualization” feature of the OTOPLAN software combines cochlear duct length estimates from the pre-operative CT scan with projected intracochlear electrode array lengths to provide individualized estimates of insertion angle and associated tonotopic frequency. Frequency estimates calculated from the organ of Corti map were preferred over the spiral ganglion map because the electrode array was being used as a recording instrument (rather than as a stimulation device) and because hair cells contribute to the acoustically evoked ECochG. Tonotopic frequency estimates combined with pre-operative audiometric profiles were used to guide the choice of electrode array length (Flex 20, 24, or 26) and insertion depth for each participant (see Table 1). The estimated tonotopic frequency from the pre-operative scan was evaluated as a factor contributing to ECochG amplitude patterns, as this estimate is available prior to surgery and can influence expectations and the planning of ECochG monitoring strategies. Pre-operative scans were performed for 10 of the 11 participants; the quality of one scan was too poor for cochlear dimension and electrode insertion depth estimates.

Estimates of electrode insertion depth and tonotopic frequency were updated using post-operative CT images, which captured the array in its actual/final intracochlear position. Post-operative scans were also used to assess the presence of scalar translocations and extracochlear electrodes. Post-operative scans were available for all participants.

Cochlear implantation was performed by one of three neurotologists using a conventional postauricular approach. The round window was accessed via the facial recess following a standard mastoidectomy. Dexamethasone was applied topically prior to opening the round window. The array was inserted via the round-window opening using a robotics-assisted insertion tool (iotaSOFT^TM^; iotaMotion Inc, Iowa City, IA, USA). Robotic electrode array insertion is slower, steadier, and more controlled compared to manual insertion [40,41,42,43]. In general, slower insertion speeds are related to better hearing preservation outcomes [44]. Reduced intracochlear trauma has been observed in human cadaveric cochleae when the iotaSOFT^TM^ tool was utilized compared to manual insertions [43]. After the array was loaded into the drive head and advanced to the cochlear entry point, speeds of electrode array insertion were set at either 0.1 or 0.2 mm/s. The back-up internal device was utilized for OP-10 due to concerns about twisting of the array after partial insertion with the first device. For OP-16, the robot was used for most of the insertion; the surgeon switched to manual insertion for the final electrodes.

Intracochlear ECochG recordings were performed with manufacturer-supplied, research-use-only software (Maestro 9.0.4 and 9.0.4.1 with Dataman Acoustic Stimulator; Med-El Corporation, Innsbruck, Austria) that controlled a MAX programming interface (reverse telemetry system to utilize the electrode array for recording) and a Dataman 531 waveform generator (acoustic stimulator). The Dataman receives a trigger from the MAX programming interface for synchronized stimulation/recording. Acoustic stimuli were routed to an ER-3C 50-Ω insert earphone with a 25-cm tube. A dome ear tip coupled to the insert earphone was placed in the ear canal of the ear to be implanted and secured prior to sterilization.

Intraoperative measurements were initiated after the surgeon reported intracochlear placement of at least one electrode. Insertion was paused to allow for an electrode impedance measurement, followed by the presentation of an electrical conditioning stimulus (500 cu) on electrodes 1 and 2 to potentially improve the intracochlear recording by further lowering the impedance. The acoustic stimulus used to elicit the ECochG was a 500-Hz tone burst (8 ms) presented with alternating polarity at 105 dB HL. The recording window was 9.6 ms. The most apical electrode (e1) was selected initially for recording.

**Figure 2:**
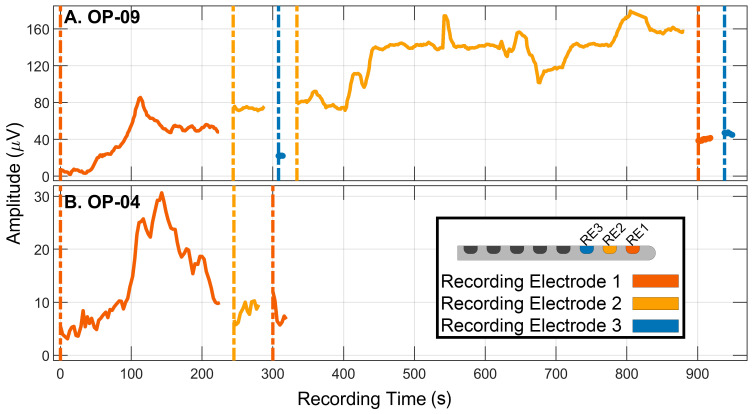
Real-time ECochG monitoring results for two participants with hearing preservation results at 500 Hz that did not match predictions from ECochG amplitude patterns. The amplitude of the first harmonic of the difference waveform is shown as a function of time. Note that although the time scale is the same for both panels, the amplitude scale has been adjusted for each data set to accommodate individual differences. Array insertion and ECochG measurements were halted each time the recording electrode was adjusted. The sections of the insertion tracks associated with different recording electrodes are marked by colored vertical lines (see also the legend). Measurements with different recording electrodes (also color-coded according to the recording electrode) are plotted sequentially within the same panel to reflect the order of events and gross timeline; however, the time between recordings was not equivalent. Additionally, electrode array advancement was not continuous during recordings but could be paused or reversed with the use of the robot. (**A**) OP-09: ECochG amplitude initially grew and peaked at 88 μV, after which amplitude declined by approximately 50%. Insertion and ECochG measurements were paused until the recording could be resumed with electrode 2. Amplitude was within 10% of the maximum measured with electrode 1. Insertion remained paused so that a recording could be made with electrode 3; amplitude was 22 μV. Electrode 2 was reselected for recording and used to monitor ECochG amplitude for the remainder of array insertion. The response grew to 160 µV. Once the array was in the final position, measurements were repeated with electrode 1 and then electrode 3. Response amplitude at these two recording locations was lower (about 40 µV) than with electrode 2. Despite the low ECochG amplitudes recorded with RE1 and RE3, the large-amplitude response at RE2 led to predictions of significant hearing preservation. OP-09 in fact showed total loss of residual hearing. (**B**) OP-04: ECochG amplitude initially grew and peaked at 30 μV during insertion, after which amplitude declined by over 50%. Four electrodes were estimated to be extracochlear at 220 s. Insertion was paused until the recording could be resumed with electrode 2. Amplitude remained low during insertion to final position. The repeat recording with electrode 1 in the final position was also low amplitude. Significant hearing loss was anticipated; however, post-operative audiometric thresholds from 125–750 Hz were within ±10 dB of pre-operative values, which is within the test-retest reliability range.

The software performed repeated fast Fourier transforms on the difference waveform (mean of 100 sweeps). Amplitudes of the first and second harmonics (i.e., the response energy contained at 500 and 1000 Hz) were updated every 20 or 10 sweeps (sliding window; proprietary weighting toward more recent sweeps). A software version change after the first three participants was responsible for the change in the number of sweeps/update speed. In the most current version, an updated amplitude estimate was available for review approximately every 1.6 s. Two examples are provided in Figure 2. The waveforms were also available for real-time review (non-overlapping averages of 100 sweeps, updated approximately every 16 s; not shown). All remaining mention of ECochG response amplitude throughout this manuscript refers to the amplitude of the first harmonic calculated from the difference waveform.

An audiologist reported the response amplitude and described the trajectory (increase, stable, decrease) in real time. References to the maximum were frequently provided for context. The surgeon integrated the feedback with multiple sources of information about the case to influence decisions such as pausing or reversing insertion or adjusting trajectory. Partial insertions were sometimes planned prior to the surgery; ECochG amplitudes could also be used to influence insertion-depth decisions.

A strategy was adopted to adjust the recording electrode if an amplitude decrement was observed. In the event of a decrement, insertion was often paused, or the electrode was slightly withdrawn to allow the recorded amplitude to stabilize. If the surgeon’s adjustments did not result in a recovered response amplitude, a new ECochG measurement was initiated with an adjacent, more basally located electrode (e2). Use of the robotic insertion system facilitated this approach, as the array remains completely stable when insertion is paused. If the ECochG amplitude was larger in the new measurement than the final amplitude in the previous recording, electrode array insertion and ECochG monitoring continued. If the same or smaller amplitude was observed at the beginning of the measurement, the next adjacent electrode in the basal direction could be tested as a recording site (e3). If an amplitude decrement was observed with the adjusted recording site, a decision could be made to switch back to e2 or e1. Time constraints limited the number of recording electrodes for any given case. If the recording electrode was adjusted during array insertion, a repeated measurement with e1 as the recording site was performed once the electrode array was in the final position.

The “final ECochG amplitude” was the last measurement that was obtained once the electrode array was in the final position (after packing around the round window and securing the lead). If the recording site had been adjusted, then a second “final” amplitude was calculated from a repeated measurement with e1. The final amplitude(s) were normalized to the maximum to quantify the global response pattern as a proportion and to facilitate comparisons across ears.

Behavioral pure-tone thresholds were used to characterize hearing sensitivity. Unaided post-operative audiometric thresholds were assessed at the initial activation appointment approximately 1-month post-operatively. These values were compared to pre-operative thresholds. If there was evidence of conductive overlay, post-operative thresholds were taken from a later time point after resolution of the conductive component. Because the ECochG monitoring stimulus was 500 Hz, preservation of hearing at 500 Hz was of interest as the matched frequency reference point. Changes in post-operative hearing relative to pre-operative hearing were quantified as percentages (modeled after Skarzynski and colleagues [45]):

(1 − [ΘPost500 − ΘPre500]/[(ML500 + 5) − Θ Pre500]) × 100(1)

where ML is the maximum limits of the audiometer (110 dB HL at 500 Hz). A “no response” at the limits of the audiometer was quantified as ML + 5 dB.

The ECochG stimulus level was suprathreshold (105 dB HL) to increase the likelihood of a sufficient response amplitude for monitoring. Due to basal spread of basilar membrane excitation, hearing sensitivity in the two-octave range above 500 Hz was also considered. Equation (1) was adapted using the frequency-specific audiometric thresholds and audiometer limits. Two hearing preservation values at 2000 Hz were greater than 100% (i.e., 150 and 200%). Upon further examination, these values came from situations where pre-operative hearing was near the audiometric limits and post-operative thresholds were better than pre-operative thresholds (but still within test-retest reliability ranges). Hearing preservation values were replaced with 100% in these cases to avoid artificially skewing the data.

ECochG amplitude patterns were quantified as the final amplitude relative to the maximum. Simple linear regression was used to test predictions of hearing preservation from final ECochG amplitude. There was no evidence that regression assumptions were violated upon examination of residuals. A linear mixed model addressed the dependency of the prediction on audiometric threshold frequency. Pre- and post-operative CT scans were evaluated against ECochG amplitude using the Spearman correlation. Statistical significance is considered *p* < 0.05.

## 3. Results

### 3.1. Participant Information

Table 1 includes demographic, device, surgical, ECochG, and hearing preservation information for the 11 participants. Ages at the time of surgery ranged from 35 to 86 years (average of 64 years). Three individuals received Flex 20 arrays, six received Flex 24 arrays, and two received Flex 26 arrays. Pre-operative hearing thresholds at 500 Hz ranged from 10 to 60 dB HL (see Figure 1). The group mean 500-Hz thresholds were 30, 37, and 43 dB HL for each respective array. The pattern is not particularly surprising because array type is not chosen at random but selected based on the individual’s pre-operative hearing coupled with cochlear anatomy. Shorter arrays tend to be chosen for individuals with greater amounts of residual hearing. Planned partial insertions and other factors such as age and etiology/prognosis also contribute to surgeon choice, which may have reduced but did not eliminate the pre-operative hearing sensitivity differences across arrays.

Insertion depth was also expected to vary across the electrode arrays with lengths of 20, 24, or 26 mm. Although shallower projected positions (and higher corresponding tonotopic frequencies) tended to be observed with shorter arrays, projected insertion depths from the pre-operative CT overlapped for the Flex 24 and 26 arrays, which illustrates the impact of cochlear size and the value of individualizing array choice. The pre-operative estimates identified two individuals (OP-04 and OP-09) for whom the projected tonotopic frequency corresponding to the place of the most apical electrode was lower than 500 Hz (the ECochG stimulus frequency). The highest pre-operative projected estimate was 1055.8 Hz for a recipient of a Flex 20 array.

All tonotopic estimates from the post-operative CT images for e1 are higher than the pre-operative estimates, which is largely explained by the number of partial insertions (extracochlear electrodes are listed in Table 1), but the same was also observed for full insertions. The lowest tonotopic estimate from the post-operative CT image was 665.3 Hz (for a partially inserted Flex 26 array); the highest was 1699.4 Hz (for a partially inserted Flex 24 array).

In 10 of the 11 post-operative CT scans, sufficient detail was available to visualize the location of electrode contacts within the cochlear labyrinth. In these 10 scans processed to assess intracochlear trauma, there were no indications of scalar translocation.

Post-operative hearing sensitivity is illustrated in Figure 1B, and hearing preservation rates for 500 Hz are provided in Table 1. Hearing preservation ranged from 0% (total loss in one participant) to 94% (a 5 dB drop for OP-17, whose pre-operative threshold was 30 dB HL). Age has previously been identified as a factor that is predictive of hearing preservation rates [21], and the tendency for older individuals to show greater loss of hearing than younger individuals was also observed in this data set. Hearing preservation rates averaged 71.5% (range of 55 to 83.3%) for the four participants aged 35 to 52 years, compared to an average of 43.4% (range of 0 to 94.1%) for the seven participants above 70 years old.

Actual ECochG amplitudes were below 200 µV for all participants except for OP-15, whose amplitude grew to 547.9 µV. This individual has a history of a vestibular schwannoma and was implanted following tumor removal. Cochlear function for this individual was likely better than suggested by audiometric thresholds given the neural contributions to the hearing loss and the large ECochG response. Because only normalized ECochG amplitudes were assessed, this individual was not excluded from the study.

### 3.2. Monitoring Strategy: Adjusting the Recording Electrode

Of the 11 participants, the recording electrode was adjusted for 6 whose ECochG amplitudes declined during the initial recording with e1. These participants were recipients of either a Flex 24 or 26 array. None of the recipients of Flex 20 arrays showed ECochG amplitude decrements >30%. Table 1 provides the list of recording electrodes used for each participant; asterisks identify the recording site with the largest amplitude once a decrement was observed with e1. The final amplitude using the adjusted, more basal electrode location was substantially larger than the final amplitude with e1 in four of the six cases using this strategy.

Figure 2 illustrates the real-time ECochG monitoring results for the two individuals with the projected e1 tonotopic position lower than 500 Hz. In both examples, amplitudes declined during the recording with e1, which was anticipated simply from the nearness of the electrode location to the traveling-wave peak. For OP-09, the amplitude recorded from e2 was larger than the previously recorded amplitude with e1, and amplitude grew as insertion continued, leading to an expectation of good hearing preservation outcomes. For OP-04, the amplitude recorded from e2 was similar to the final recording with e1, leading to an expectation of poor hearing preservation outcomes. These participants will continue to be highlighted in subsequent sections.

### 3.3. ECochG Amplitude Decrements as a Predictor of Hearing Preservation

**Figure 3:**
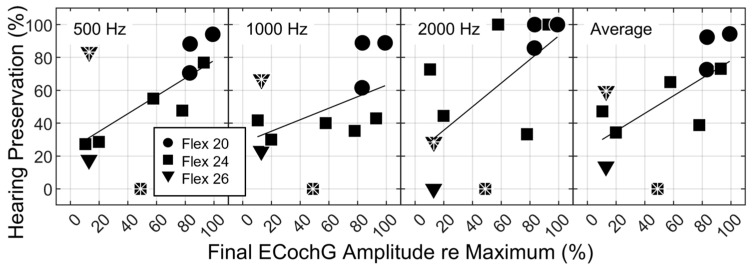
Hearing preservation as a function of the normalized final ECochG amplitude recorded at e1. Panels are labeled with the frequency at which hearing preservation was assessed. The ECochG stimulus was always 500 Hz. Symbol denotes electrode array type (see legend). OP-04 and OP-09 are marked with asterisks. Simple linear regression results for 500 Hz and the average are provided in Table 2.

Hearing preservation is plotted as a function of final ECochG amplitude (converted to a percentage of the maximum amplitude) in Figure 3. The data from OP-04 and OP-09 are marked with asterisks.

A primary aim of this study was to evaluate whether considering recordings from more basal electrode sites could be used to improve real-time predictions of trauma/post-operative hearing preservation. For OP-09, a larger ECochG response was observed when recording from e2 than from e1. The hypothesis was that recording a larger response from a more basal site would support an interpretation that the amplitude decrement observed with e1 was atraumatic. OP-09 experienced total hearing loss, meaning the revised prediction of hearing preservation using e2 was even worse than the original prediction with e1. For OP-04, a similarly small amplitude was recorded from e1 and e2. The hypothesis was that a similar, small amplitude at a basal site would support an interpretation that the amplitude decrement observed at e1 was the result of a traumatic event. Hearing loss was anticipated for OP-04, and yet post-operative hearing preservation was better than expected, particularly at low frequencies. Post-operative hearing sensitivity at 125, 250, and 500 Hz was within the test-retest reliability range. Simple linear regression, when considering the use of ECochG decrements to predict hearing preservation at 500 Hz, was repeated by replacing the e1 amplitude decrements with those from the adjusted recording electrode site for six participants (data are not shown in Figure 3 but are included in Table 1). The r^2^ value decreased from 0.36 to 0.02, further demonstrating that the recording electrode strategy did not improve predictions (Table 2; top two rows).

The second aim of the study was to evaluate whether hearing preservation predictions from the ECochG would be improved when including hearing sensitivity at frequencies higher than the ECochG stimulus. This prediction was based on the use of a suprathreshold, high-stimulus level to evoke the ECochG and the spread of excitation basal to the characteristic frequency place from the traveling wave tail.

To evaluate the impact of higher frequencies on hearing preservation, we implemented a linear mixed model with fixed effects of frequency (500, 1000, and 2000 Hz) and ECochG amplitude decrements, as well as the interaction. The interaction was the primary comparison of interest. A random intercept for the participant was included to account for repeated measures across frequency. The intraclass correlation was 0.71, indicating substantial correlation of hearing preservation between frequencies. The interaction (*p* = 0.28) indicates that the relationship between ECochG amplitude decrements and hearing preservation does not vary by frequency. Neither the main effect of frequency nor ECochG amplitude decrements was statistically significant (*p* = 0.89 and *p* = 0.06, respectively). These results suggest that hearing preservation predictions do not improve by considering hearing sensitivity at frequencies higher than 500 Hz compared to predictions focusing on 500 Hz.

Since the results do not differ across discrete frequencies, simple linear regression was repeated using hearing sensitivity averaged across 500, 1000, and 2000 Hz as the dependent variable. Those results are provided in Table 2. Despite a *p* value < 0.05, there is only a 2% difference in the amount of variance explained by the ECochG decrement when considering the average compared to hearing preservation only at 500 Hz. Taken together, the results do not provide strong evidence supporting the predictions of Aim 2.

### 3.4. The Impact of Recording Site on ECochG Amplitude

**Figure 4:**
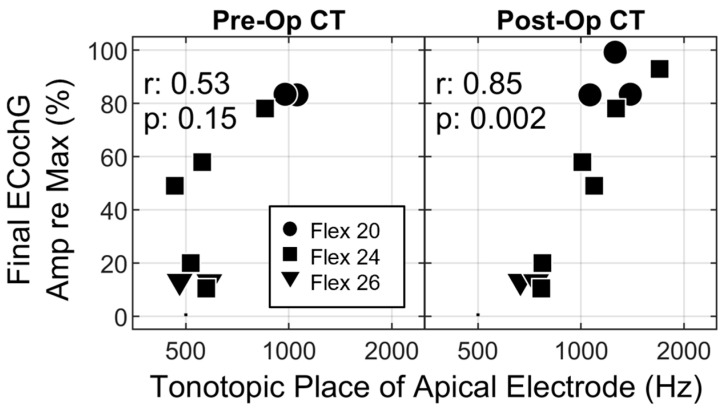
Scatterplots relating ECochG amplitude and the intracochlear location of the recording electrode (e1) estimated from pre- (**left**) and post- (**right**) operative CT scans. Symbols denote array type (see legend). Spearman correlation coefficients and *p* values are provided in each panel.

The final aim of this study was to further characterize the impact of the intracochlear location of the recording electrode on ECochG measures. Both pre- and post-operative estimates of electrode location were evaluated (see Figure 4). Pre-operative estimates were of interest since they are available at the time of surgery and could potentially be integrated into a real-time interpretation of the ECochG. Post-operative estimates reflect the actual/final position of the electrode array.

A significant positive correlation was observed between tonotopic location and the ECochG amplitude decrement. This correlation was moderate (r = 0.53) although not statistically significant when considering pre-operative estimates, but it was significant with a high correlation (r = 0.85) when using the position estimated from the post-operative scan. This correlation is stronger than the relationship between ECochG amplitude and hearing preservation outcomes (Figure 3). Of particular interest is that the data for OP-04 and OP-09 follow the predicted pattern. These results raise the possibility that electrode location seems to explain why hearing preservation results were not predictable from the ECochG patterns for these two cases.

## 4. Discussion

This study expands upon previous investigations of ECochG for real-time monitoring by (1) evaluating whether adjusting the recording electrode would improve predictions of post-operative hearing loss from the ECochG amplitude decrements; (2) considering hearing sensitivity at frequencies higher than the ECochG stimulus frequency; and (3) illustrating the confounding effect of the intracochlear electrode position on the interpretation of single-frequency ECochG amplitude decrements. Neither adjusting the recording electrode nor including behavioral thresholds at 1000 and 2000 Hz improved predictions of post-operative hearing sensitivity. The intracochlear position of the recording electrode was more strongly correlated with ECochG amplitude decrements than the correlation between ECochG amplitude and post-operative hearing preservation.

### 4.1. Real-Time Recording Electrode Adjustments

Selecting adjacent electrodes in succession was anticipated to be a sufficient recording site adjustment given the length of the Flex 24 and 26 arrays and spacing across electrodes (1.74 and 1.9 mm, respectively). Although maximal ECochG responses evoked with a 500-Hz stimulus have been recorded up to six electrodes away from the most apical site for modiolar-hugging arrays, the maximum response has been observed primarily at the apical electrode for lateral wall arrays [25]. A single case involving a Cochlear Slim 20 array where a maximum was recorded at a more basal site (electrode 20 of 22) occurred for the subject with the deepest insertion angle (385°) [25]. MedEl Flex arrays are designed for lateral wall placement, and the 24- and 26-mm arrays have a similar anticipated insertion depth compared to the Cochlear Slim 20 [4,46]. Insertion angles for the subjects in this study with Flex 24 and 26 arrays ranged from 267.6° to 422.1° (mean 359°); three arrays were positioned at deeper insertion angles than the case highlighted by Walia and colleagues [25]. The spacing between electrodes 20 and 22 for the Cochlear Slim 20 array is similar to the spacing between adjacent electrodes for the Flex 24 and 26 arrays. With the exception of OP-04, electrode 3 was also evaluated as a recording site, which is 3.48 to 3.8 mm from the apical electrode. Time constraints limited the number of recording electrodes that could be evaluated in real time, so it is unknown whether recording from a site basal to e3 (or e2 in the case of OP-04) would have improved the interpretation of amplitude fluctuations. The ability to record simultaneously from multiple electrodes, to interleave recordings from multiple intracochlear electrodes throughout the advancement of the array, or simply to modify the recording electrode within the same monitoring window would be welcome additions to commercial systems.

### 4.2. Basalward Spread of the Traveling Wave at High Stimulus Levels

Given the high stimulus level and low frequency used to elicit the ECochG, it was of interest to determine whether amplitude decrements would better predict hearing sensitivity when including frequencies higher than the ECochG stimulus. This prediction was based on the general notion that the traveling wave broadens with stimulus intensity, and the peak shifts to a more basal site [47]. This mechanical pattern is reflected in amplitude measurements of the cochlear microphonic [48]. The distance between the point of maximum sensitivity (the threshold measurement) and the point of maximum voltage (at a high stimulus level) is approximately 4 mm. For context, the place of maximum excitation across frequency (i.e., tonotopic organization) shifts about 2 mm per octave [48].

In the present investigation, including audiometric thresholds at 1000 and 2000 Hz (one and two octaves above the ECochG stimulus) in the hearing preservation calculation did not improve predictions from the ECochG (Table 2). A complicating factor is that, despite using a constant stimulus level for eliciting the ECochG across participants, they had varying degrees of hearing loss and residual hair cell function. The impact of the spatial extent of residual cochlear function and interaction with the stimulus level on potential basal contributions to the ECochG is unknown.

### 4.3. Intracochlear Location of the Recording Electrode

A relationship between recording electrode insertion depth and ECochG amplitude decrement was anticipated given that ECochG maxima are more frequently measured at basal electrodes for arrays with deeper insertion angles [25]. This study illustrates the linearity of the relationship and quantifies the strength.

In this data set, ECochG amplitude decrements were smallest for electrodes that terminated between the 1000 and 2000 Hz tonotopic place, or approximately 3 mm basal to the expected tonotopic site for 500 Hz. Though this finding is a cross-subject observation, it is generally consistent with the post-operative measurements performed across multiple electrodes for a given individual: the recording electrode with the maximum amplitude tends to be more basal than anticipated from the tonotopic map [49,50]. This result is also consistent with observations that the cochlear microphonic component appears dominated by contributions of the tails of the excitation due to phase cancellations at the peak [34].

None of the apical electrodes crossed the 500 Hz place, largely because of the number of partial insertions, and yet ECochG amplitude declined to 10–22% of the maximum in four cases. An explanation that has become popular to describe an atraumatic amplitude decrement is the advancement of the recording electrode past the generation site. The tonotopic site of maximal sensitivity was not passed by the apical electrode in any of the cases included in this report. OP-04 specifically is an example of an atraumatic decrement. These results suggest that atraumatic amplitude decrements can be observed even for electrodes that terminate basal to the tonotopic place of the ECochG stimulus.

### 4.4. Partial Insertions

The number of partial insertions in this data set was high (7 of 11 participants). The shallowest insertion was 267.6°, which is similar to the average insertion depth for a L24 array [4]. This center has observed positive outcomes not only for recipients of the L24 array [51], but also 10 mm-long arrays with fewer (6–10) intracochlear electrodes [52,53]. These positive experiences combined with evidence that trauma risk increases with insertion depth [54] influence decisions surrounding planned partial insertions. Although this study did not specifically include outcome measures of benefit (e.g., speech perception), the ultimate motivation when pursuing cochlear implantation is to provide patients with the best possible functional hearing outcomes. The benefits from combining electric and acoustic signals are well established [52]. An obvious concern arises when acoustic hearing is lost, but the same study showed that bimodal (contralateral acoustic hearing and ipsilateral electric hearing) performance was comparable between individuals with short arrays who lost acoustic hearing compared to individuals with standard-length arrays [52]. For all individuals, but particularly those who lose more residual hearing than anticipated (e.g., OP-09), outcomes are carefully monitored and influence subsequent discussions about ongoing clinical care. A clinical plan following loss of residual acoustic hearing can include revision surgery, but revision is not advised in all cases.

### 4.5. Real-Time Feedback

Few studies have investigated the provision of real-time feedback on hearing-preservation outcomes. Harris and colleagues [55] used an audible signal that was steady, rising in pitch during ECochG amplitude increases, or “knocking” when amplitude declined by 6 dB (i.e., half) to alert the surgeon to pause and begin adjustments. Hearing preservation outcomes for the control group were no different than the group for whom feedback was provided. In contrast, a more successful effect of feedback was observed by Bester and colleagues [56]. Changes in post-operative hearing sensitivity were smaller in cases where the ECochG declined but subsequently recovered following surgical adjustment, compared to cases where no recovery was observed. Notably, the cutoff for alerting the surgeon was more conservative (i.e., amplitude decrement >30% by visual judgement), and a more standardized approach to adjusting the array position was utilized. In the present investigation, real-time feedback was provided for all participants, and therefore we cannot assess whether hearing preservation outcomes were positively impacted by incorporating ECochG into the surgical procedures.

### 4.6. Limitations

The slow speed at which physiological information is updated limits the effectiveness of potentially corrective actions. In this study, the real-time ECochG analysis updated every 1.6 sec with an amplitude that was the moving average of previous sweeps (though weighted to most recent sweeps). Although some systems have automated alerts, this study used an audiologist to relay information to the surgical team. The processing speed and communication decisions of the intermediary further delayed the transmission of information. In this investigation, the slow provision of feedback was partially mitigated by the use of the robot that controlled electrode array insertion speeds at a slower rate than can be achieved manually and that could be stopped in a stabilized state at any moment. An insertion device that integrates ECochG directly would be reduce the time delay between an electrophysiological change and a reaction, likely improving the effectiveness of surgical modification [57].

This study simplified the ECochG amplitude pattern by focusing on the final amplitude relative to the maximum. In some instances, multiple decrements were observed. For example, a decrement occurred in the middle of insertion for OP-01 and resolved by the end of insertion. By focusing on final amplitude, decrements such as these were not captured but are likely relevant when considering the risk of intracochlear trauma across the entire procedure [35].

This study was also limited to a single metric: the amplitude decrement of the ECochG evoked with a single frequency. Numerous analytical opportunities exist with the ECochG, and the ideal metric for real-time monitoring is likely a combination of several measures [25,30,35,36,37,38]. Automated, objective algorithms will be necessary to integrate across various metrics with sufficient speed to provide useful feedback to the surgical team.

Due to the small sample size, the analyses reported within this manuscript were necessarily limited. Multiple factors contribute both to ECochG response amplitude patterns and to hearing preservation, and these factors likely co-vary. For example, pre-operative hearing was better for shorter arrays than longer arrays in this sample; shorter arrays were associated with shallower insertion depths, larger ECochG amplitudes, and better hearing preservation post-operatively. It is uncertain which factors are causative and which are the consequence, or how they interact. More work is needed with larger samples to better understand the contributions of the multiple factors.

## 5. Conclusions

Because CI recipients, especially those considered for electro-acoustic stimulation management, tend to have limited residual hearing at high frequencies, ECochG monitoring is often restricted to low frequencies. The likelihood of atraumatic ECochG amplitude fluctuations increases as the recording electrode advances. The risk of intracochlear trauma also increases with insertion depth [54]. It is crucial to separate atraumatic from traumatic ECochG amplitude decrements to provide the most useful feedback to the surgical team for making decisions regarding electrode array positioning. Despite some association between ECochG amplitude decrements and post-operative hearing preservation, the intracochlear location of the recording electrode confounds interpretation.

## Figures and Tables

**Table 1:** Participant information, ordered by estimated tonotopic frequency associated with the projected location of the apical electrode (e1) from the pre-operative scan (assumes full insertion).

ID	Age (Years)	Ear	Etiological Considerations	Onset	Flex Array	e1 Tonotopic Frequency (Hz)		e1 Insertion Angle (Degrees) Post-Op	Extracochlear Electrodes	RE	Final ECochG Amplitude (% max)		500-Hz HP (%)
						Pre-Op	Post-Op				RE1	RE*	
09 †	79	L	Noise	Adult	24	463.9	1093.4	331.9	10 ▪,11,12	1, 2 *, 3	22	86	0
04 †	35	L	Idiopathic	Teenager	26	479.4	665.3	422.1	12	1, 2 *	13	23	83
16	86	R	Idiopathic	Adult	24	516.6	772.2	394.5	12 ▪	1, 2, 3 *	20	98	29
03	41	R	Idiopathic	Early Adult	24	557.8	1010	347.3	11,12	1,2 *, 3	58	36	55
10	72	L	Noise	Adult	24	574	765.4	393.9	11 ▪,12	1, 2 *, 3	10	76	27
08	72	L	Idiopathic	Adult	26	585.7	739.1	403	None	1, 2, 3 *	13	75	18
07	78	R	Noise, Family History	Adult	24	852.8	1264.4	311.8	12	1	78		48
02	71	L	Suspected Genetic	Childhood	20	975.2	1393.6	296.5	None	1	83		88
01	48	L	Idiopathic	Teenager	20	1055.8	1062.3	383.3	None	1	83		71
17	74	L	Noise	Adult	20	poor scan	1258.3	321.1	None	1	99		94
15	52	L	Vestibular schwannoma	Adult	24	no scan	1699.4	267.6	10, 11, 12	1	93		77

ID: identifier; L: left; R: right; e1: electrode 1 (apical); Hz: hertz; Pre-Op: pre-operative; Post-Op: post-operative; RE: recording electrode(s); HP: hearing preservation. † Marks two subjects with an estimated tonotopic frequency location of the apical electrode lower than the 500 Hz characteristic frequency place. ▪ Indicates electrode at the round window. * Denotes which electrode was selected as the adjusted site following an amplitude decrement observed at e1.

**Table 2:** Simple linear regression results for predicting hearing preservation from intra-operative ECochG monitoring (final amplitude relative to the maximum).

ECochG Recording Site	Hearing Preservation Frequency	Intercept (*p*-Value)	Slope (*p*-Value)	r^2^
RE1	500 Hz	24.13 (0.15)	0.54 (0.05)	0.36
RE*	500 Hz	66.36 (0.08)	−0.17 (0.70)	0.02
RE1	Average of 500, 1000, 2000 Hz	24.50 (0.12)	**0.54** **(0.04)**	0.38

RE1: recording from electrode 1; RE*: recording from the adjusted site (e2 or e3). Results with *p*-values < 0.05 are bolded.

## Data Availability

The data presented in this study are openly available in Open Science Framework at DOI 10.17605/OSF.IO/638US.
